# Relative United States Medical Licensing Examination (USMLE) Performance by Specialty Is Not a Predictor of Board Exam Pass Rate: The Case of Diagnostic Radiology

**DOI:** 10.7759/cureus.12725

**Published:** 2021-01-15

**Authors:** Surav M Sakya, Mary L Dinh, Donald Chan, Cory M Pfeifer

**Affiliations:** 1 Medicine, Penn State College of Medicine, Hershey, USA; 2 Internal Medicine, Riverside Community Hospital, Riverside, USA; 3 Radiology, University of Texas Southwestern Medical Center, Dallas, USA

**Keywords:** usmle, diagnostic radiology, board exam, residency education

## Abstract

Introduction

In 2010 diagnostic radiology (DR) changed the board certification process for residents using the new Core exam. However, there is not a standardized way to evaluate DR residency graduates. With no specific target pass rate for the exam, the “appropriate” pass rate has remained a debated topic among the field. In this paper, the board certification exam passage rates of DR are compared to other medical specialties to assess the standardization method of the American Board of Radiology (ABR) and serve as basis for additional specialties considering changes to their board exam structure.

Methods

Performance on the United States Medical Licensing Examination (USMLE) was obtained from the National Resident Matching Program (NRMP) and San Francisco match. Boards passage rates were analyzed using data from the American Board of Medical Specialties. USMLE and board exam passage rates were averaged and ranked, and statistical analysis was conducted using Stata (College Station, TX).

Results

DR performance on USMLE Step 1 has increased at the lowest rate (0.563 points/year) since 2005 and anesthesiology performance has increased at the greatest rate (1.313 points/year). Residents matching from US allopathic medical schools during the 2010 and 2012 years had DR oral board exams with USMLE 1 averages of 232 and 235, respectively. First-time pass rate for the first Core exam was 87% and the overall pass rate since the first Core exam has been 88.54%. The Spearman rho coefficient for specialty ranks of board passage rate and USMLE 1 was 0.0679 (p = 0.8101). The Spearman rho coefficient for board passage rate and USMLE 2 CK was 0.1430 (p = 0.6257). The Spearman rho coefficient for USMLE 1 and USMLE 2 CK was 0.8317 (p = 0.0002).

Conclusions

Specialty board pass rates have not increased in concert with improved trainee performance on the USMLE. USMLE performance among those matching in diagnostic radiology has increased, ABR board exam passage rate has decreased. ABR determines passing thresholds to the relative performance of examinees rather than using a criterion referenced Angoff standard.

## Introduction

Diagnostic radiology (DR) has had competitive applicants over the past 10 years [1], and the United States Medical Licensing Examination (USMLE) Step 1 average among matched US graduates held steady at 241 in 2020 [2]. With that said, the fill rate has fluctuated from 99% in 2009 [3] to 92% in 2012 [4] followed by an upward trend to 99% in 2018 [5].

Following a 2006 nationwide survey of practicing radiologists, the American Board of Radiology (ABR) revamped the board certification process for residents beginning radiology residency training in 2010 [6]. The new Core exam would be standardized using the Angoff method whereby subspecialty experts would determine the minimum competency for each section [7]. As such, no specific target pass rate for the exam was advertised. It would be therefore theoretically possible for all or none of the residents taking the exam to pass. Questions were raised as to how ABR experts would determine this competency level for residents with greater than a quarter of residency training (residency training is four years) remaining in contrast to the former oral exam given at the end of training, and some worried that smaller programs would be at a disadvantage in the new system [8].

Due to the increase in competition for DR residency slots in 2009, the first group of residents to take the then-new ABR Core exam in 2013 were among the highest achieving cohort of residents that DR had ever trained. Nonetheless, the ABR Core exam pass rate mirrored the pass rates of prior ABR board certification examinations [9]. Despite the promise of the ABR that this new exam would not place smaller programs at a disadvantage [7], chief resident-derived data obtained after the first two administrations of the Core exam suggested that small program size was indeed a risk factor for failing the Core exam [10].

The “appropriate” pass rate has remained a debated topic among trainees and program officials. This paper seeks to compare the board certification exam passage rates of DR to other medical specialties to assess the standardization method of the ABR and serve as basis for additional specialties considering changes to their board exam structure.

## Materials and methods

Performance on the USMLE was obtained from the National Resident Matching Program (NRMP) and San Francisco match [11]. This is reported in Table 1 and Table 2 with specialties grouped according to the ACGME designations of primary care, hospital-based, and surgical specialties.

**Table 1:** Average USMLE 1 scores of United States allopathic medical school graduates by specialty. *Certain specialties have unreported USMLE scores as represented by empty spaces. USMLE: United States Medical Licensing Examination

Specialty*	2005	2007	2009	2011	2014	2016	2018	2020	
Dermatology	233	238	242	244	247	249	249	248	
Family Medicine	210	211	214	213	218	221	220	221	
Internal Medicine	220	222	225	226	231	233	233	235	
Neurology		219	225	225	230	231	231	232	
Pediatrics	215	217	219	221	226	230	227	228	
Physical Medicine and Rehabilitation	208	209	214	214	220	226	225	228	
Psychiatry	210	210	216	214	220	224	226	227	
Anesthesiology	213	220	224	226	230	232	232	234	
Diagnostic Radiology	232	235	238	240	241	240	240	241	
Emergency Medicine	219	220	222	223	230	233	233	233	
Pathology	222	223	227	226	231	233	233	233	
Radiation Oncology	228	235	238	240	241	247	247	243	
General Surgery	222	222	224	227	232	235	236	237	
Neurological Surgery			239	239	244	249	245	248	
Obstetrics and Gynecology	212	214	219	220	226	229	230	232	
Ophthalmology	229	231	235	237	242	244	245	245	
Orthopedic Surgery	230	234	238	240	245	247	248	248	
Otolaryngology		238	240	243	248	248	248	248	
Plastic Surgery	231	241	245	249	245	250	249	249	
Vascular Surgery					237	239	236	239	

**Table 2:** Average USMLE 2 CK scores of United States allopathic medical school graduates by specialty. *Certain specialties have unreported USMLE scores as represented by empty spaces. USMLE: United States Medical Licensing Examination

Specialty*	2007	2009	2011	2014	2016	2018	2020
Dermatology	242	251	253	255	257	256	256
Family Medicine	218	223	225	234	237	237	238
Internal Medicine	227	232	237	243	246	246	248
Neurology	223	231	233	241	243	242	245
Pediatrics	225	229	234	241	244	243	245
Physical Medicine/Rehabilitation	214	220	224	234	238	239	241
Psychiatry	213	221	225	233	238	239	241
Anesthesiology	223	230	235	241	242	244	246
Diagnostic Radiology	237	242	245	249	247	249	249
Emergency Medicine	227	230	234	243	245	247	247
Pathology	226	230	233	241	243	242	242
Radiation Oncology	237	241	244	248	251	253	250
General Surgery	226	231	238	245	247	248	249
Neurological Surgery		237	241	247	251	249	252
Obstetrics and Gynecology	223	229	233	242	244	247	248
Orthopedic Surgery	235	241	245	251	253	255	255
Otolaryngology	241	246	250	252	253	254	256
Plastic Surgery	244	245	249	252	256	254	256
Vascular Surgery				250	250	244	247

Data is only available from programs participating in the NRMP or San Francisco match as of the year noted. Ophthalmology does not report USMLE 2 Clinical Knowledge (CK) averages. The American Urology Association publishes data detailing applicant totals and fill rates but does not publish USMLE data.

Most American Board of Medical Specialties (ABMS)-participating boards publish passage rates for their board exams. The website of each ABMS board was accessed in 2016, 2017, 2018, and 2019, and the available data is reported in Table 3.

**Table 3:** Percent passing of exams given by ABMS-participating boards. *Certain specialties have unreported pass rates as represented by empty spaces. ABMS: American Board of Medical Specialties

Specialty*	2005	2006	2007	2008	2009	2010	2011	2012	2013	2014	2015	2016	2017	2018	2019	2020
Allergy and Immunology		93	88	90	91	91	96	97	89	94	93	92	82	83	83	
Anesthesiology Part 1				86	92	85	87	90	87	90						
Anesthesiology Part 2				85	81	84	88	87	88	88						
Anesthesiology Basic										96.5		91	88.4			
Anesthesiology Advanced												94	95.2			
Diagnostic Radiology Physics	87	90	87	90	90	94										
Diagnostic Radiology Clinical	91	95	96	98	97	91	98	94								
Diagnostic Radiology Oral	86	85	90	88	90	92	89	89								
Diagnostic Radiology Core									87.2	91	86.8	91.1	93.5	86.2	84	
Emergency Qualifying	90	92	90	91	91	91	91	94	89	90	91	93	93	95	92	
Emergency Oral	95	94	94	95	95	94	97	98	98	96	98	98	96	97	95	
Family Medicine	96.7	97	85.4	87.8	88.3	86.6	85.1	85.5	87.1	91	95.6	97.8	98.1	98.7	98.6	
General Surgery Qualifying							80	81	79	79	80	80	90	97	96	
General Surgery Certifying							76	72	80	78	77	80	79	80	85	
Internal Medicine							84	85	86	87	89	90	90	91	91	
Medical Genetics/Genomics	87.6		91.2		86.8		91.7		89.4		92		91		91	
Neurology									87	87	90	88	86	88	88	
Obstetrics/Gynecology Written								92								
Obstetrics/Gynecology Oral								84								
Ophthalmology Written																
Ophthalmology Oral Spring					88.21	83.3	84.9	84.9	90.1	86.11	90.4	87.2	88.7	93.1	97.8	
Ophthalmology Oral Fall				82.96	84.21	78.41	84.82	79.48	81.03	83.81	81.47	72.5	80.3	87.4	85.8	
Orthopaedic Surgery Part I				82.87	80.09	79.53	82.67	86.76	81.92	82.92	82.06	77.9	83.1	85.3	78.3	
Orthopaedic Surgery Part II							89	94	93	95	96	96	97	97	97	
Pathology-Anatomic										91						
Pathology-Clinical										90						
Pediatrics						76	76	86	82	87	86	81	88	91	87	
Physical Med/Rehab Written											86		93.73		94.6	
Physical Med/Rehab Oral											80		89.16		96.9	
Preventive Medicine	78	75	80	76	71	70	74	77	72	92	94	84	82	83	83	
Psychiatry									87	90	88	90	89	87	88	
Radiation Oncology Clinical	89	95	95	98	98	96	94	95	93	92	97	95	95	97	93	
Radiation Oncology Physics	84	92	85	95	89	90	96	80	91	81	98	97	92	71	98	
Radiation Oncology Biology	92	98	95	96	96	91	97	88	96	87	89	94	90			
Radiation Oncology Oral	86	90	86	80	89	85	82	82	89	93	88	90	90	92	88	
Thoracic Surgery Written						81	87	85	85	81	82	86	86	95		
Thoracic Surgery Oral							66	70	76	72	72	78	84	77		
Urology Qualifying	90	88	91	88	91	90	90	93	93	94	94	97	99	97	98	
Urology Certifying	95	93	91	92	93	94	92	89	91	91	86	94	92	94	96	94

This represents pass rates from first-time exam takers. Some data discoverable as of 2016 did not continue to be publicly reported in subsequent years.

USMLE and board exam passage rates were averaged and ranked. This is shown in Table 4.

**Table 4:** Mean USMLE scores of matched US allopathic medical school graduates and board exam pass rates. N/A = Not Available * = Tie USMLE: United States Medical Licensing Examination

Specialty	Pass Rate (Rank)	USMLE 1 (Rank)	USMLE 2 CK (Rank)
Emergency Medicine	93.62 (1)	226.63 (9)	239 (6)
Orthopaedic Surgery	93.19 (2)	241.25 (1)	247.86 (1)
Radiation Oncology	91.17 (3)	239.88 (2)	246.29 (2)
Family Medicine	90.92 (4)	216 (15)	230.29(12)
Diagnostic Radiology	90.85 (5)	238.38 (4)	245.43 (3)
Pathology	90.5 (6)	228.5 (6)	236.71 (11)
Psychiatry	88.8 (7)	218.38 (13)	230 (13*)
Anesthesiology	88.58 (8)	226.38 (10)	237.29 (9)
Obstetrics and Gynecology	88 (9)	222.75 (12)	238 (7)
Neurology	87.6 (10)	227.57 (8)	236.86 (10)
Internal Medicine	87.29 (11)	228.13 (7)	239.86 (5)
Physical Medicine and Rehabilitation	87.22 (12)	218 (14)	230 (13*)
Ophthalmology	83.65 (13)	238.5 (3)	N/A
Pediatrics	82.75 (14)	222.88 (11)	237.29 (8)
General Surgery	80.25 (15)	229.38 (5)	240.57 (4)

Each specialty was ranked by board passage rate, USMLE 1, and USMLE 2 CK. Spearman rho coefficients and associated p values were calculated using Stata (College Station, TX).

## Results

USMLE performance has improved since 2005 across nearly all specialties. Of those specialties with continuous participation, DR performance on USMLE Step 1 has increased at the lowest rate (0.563 points/year) since 2005 while anesthesiology performance has increased at the greatest rate (1.313 points/year).

Residents matching from US allopathic medical schools taking the 2010 and 2012 DR oral board exams had USMLE 1 averages of 232 and 235, respectively. Residents matching from US allopathic medical schools taking the first DR Core exam scored an average of 238. Despite the rising performance on USMLE 1 and USMLE 2 CK, passage rates on the DR Core exam dropped at all-time low in 2019.

The first-time pass rate for the first Core exam was 87% [12], and the overall pass rate since the first Core exam has been 88.54%. Of the first-time pass rates for DR exams published by the ABR since 2005, the average pass rate has been 90.459% with the last six DR Physics exams averaging 89.67%, the last eight DR Clinical (commonly referred to as the “old written” exams) averaging 95%, and the last eight DR oral exams averaging 88.625%.

The Spearman rho coefficient for specialty ranks of board passage rate and USMLE 1 was 0.0679 (p = 0.8101). The Spearman rho coefficient for board passage rate and USMLE 2 CK was 0.1430 (p = 0.6257). The Spearman rho coefficient for USMLE 1 and USMLE 2 CK was 0.8317 (p = 0.0002).

## Discussion

As specialty boards seek to respond to advances in testing technology, greater exam preparation resources, and the need improved standardization, they are faced with challenges if they change the format of time-tested examination methods. Much attention has been paid to changes in the Initial Certification process in Diagnostic Radiology within the greater climate of ACGME and ABMS evolution, as it switched from an oral examination to the computerized Core and Certifying exams starting with the graduating class of 2014.

The first query in any transition focuses on whether a novel test or process retains the same content validity as in the prior setting and whether criterion validity has been sacrificed for simplicity. In describing the new board examination process in 2013 [7], the ABR answered the rumor that “10% of Board examinees must fail exams” by explaining that a panel of experts determines the level of competency commensurate with safe practice regardless of how many examinees fail as a result. The oral system, in contrast, utilized a panel of experts who assessed the candidate on a face-to-face basis. The evidence basis for why the new system is more effective than the former system remains a source of debate. In fact, the 2014/2015 program directors’ survey revealed that “91% felt that the ABR Oral Examination was superior to the Core Examination in testing readiness for clinical practice” [13].

Several conclusions can be drawn from these data. First, while there is no “standard” acceptable fail rate in any specialty, the 10% suggested by Becker et al. is not far from reality [7]. Second, there is no correlation between rates of board exam pass rate and USMLE I performance even though there are clear disparities between USMLE I performance in each specialty. If one were to take USMLE I as a relative measure of one’s ability to perform well on medical multiple choice exams among a pool of exceptional learners, this suggests that each specialty sets a standard within the pool of physicians that they have, not the total population of medical graduates. Third, as performance on the medical licensing exams has shown an upward trend, board passage rates have not followed in concert. Relevant to this discussion is the fact that the USMLE has not changed its scoring system as examinees improve. USMLE score inflation is a clear example of the effect of exceptional test takers availing themselves of improved exam resources over time, and the overall question of how generalizable the application of standard psychometric testing procedures will continue to apply to examinees of remarkable intellect in an era of ever-expanding resource material will persist.

If the core values of a board certifying body include public trust, it may be reasonable to admit that not all takers should pass lest the credibility of the board be at risk. A system in which a 100 percent pass rate is typical would suggest that the responsibility to verify the acumen of the specialty’s practitioners would lie with the training programs and not the board. This would be counter to the board’s mission. The fact that the first Core exam fail rate mirrored the average pass rate across all specialties and the rates of prior ABR exams adds to the perception of validity. Regardless, using any psychometric process to exclude items in an attempt to discriminate between those who pass and those who fail assumes that there will be candidates who fail.

The usage of recalled examination items by takers of the prior clinical exam was discussed by Berlin in 2012 [14] and Ruchman et al. in 2008 [15]. Though the practice of sharing ABR examination content is now more clearly forbidden, and the current Core exam has reportedly reduced the number of reused items, a valid concern regarding reliability is raised. If the reuse of exam items improves the ability to equate prior administrations with current administrations as described by the ABR, it becomes impossible to know whether a given examinee knows the correct answer to a reused question because of comprehensive preparation or because he or she was told the item would be tested. If a passer is defined as one who performs well on validated discriminatory reused questions, an obvious bias emerges in favor of the utilizer of contraband recalled items.

The decision to force examinees to take the Core exam at the end of PGY-4 was likely related to the intent to deemphasize any possible “recall” advantage generated by examinees who violate ABR policy, but it also begs the question as to whether there is a benefit to forcing a resident to take this high-stakes exam before he or she feels adequately prepared. Self-reported data from the first two administrations of the Core exam [10] suggested that easing of clinical duties near the exam was a negative predictor of success. It seems reasonable that a program may desire to hold select residents back several months if it seems as though clinical experience may be insufficient.

As for the autumn administration of the Core exam, the candidate pool as it is now must almost entirely consist of alternate certification applicants - many of whom have completed a residency outside of the United States in addition to multiple fellowships - and PGY-5 residents who have failed the exam at least once. From an onlooker’s perspective, it seems nearly impossible to compare the results of such a sample to the traditional candidate pool taking the exam in the spring administration. Allowing first-time traditional examinees into this pool may improve the ability to ensure that the exam is uniform between both administrations.

## Conclusions

Specialty board pass rates have not increased in concert with improved trainee performance on the USMLE. Specialty ranks according to USMLE 1 and USMLE 2 CK are statistically similar, however, neither USMLE 1 nor USMLE 2 CK ranks correlate with board passage rate. While USMLE performance among those matching in diagnostic radiology has increased, ABR board exam passage rate has declined. The data presented here suggests that the ABR determines passing thresholds to the relative performance of examinees rather than using a criterion referenced Angoff standard.
